# Four chemical methods of porcelain conditioning and their influence over bond strength and surface integrity

**DOI:** 10.1590/2176-9451.20.4.051-056.oar

**Published:** 2015

**Authors:** João Paulo Fragomeni Stella, Andrea Becker Oliveira, Lincoln Issamu Nojima, Mariana Marquezan

**Affiliations:** 1 Professor, Centro de Estudos Odontológicos Meridional (CEOM), Postgraduate program, Passo Fundo, Rio Grande do Sul, Brazil; 2 Professor, Universidade Federal do Rio de Janeiro (UFRJ), Department of Pediatric Dentistry and Orthodontics, Rio de Janeiro, Rio de Janeiro, Brazil; 3 Postdoc resident, Universidade Federal do Rio de Janeiro (UFRJ), Department of Pediatric Dentistry and Orthodontics, Rio de Janeiro, Rio de Janeiro, Brazil. Dentist, Universidade Federal de Santa Maria (UFSM), Department of Restorative Dentistry, Santa Maria, Rio Grande do Sul, Brazil

**Keywords:** Orthodontic brackets, Ceramics, Orthodontics

## Abstract

**OBJECTIVE:**

To assess four different chemical surface conditioning methods for ceramic material before bracket bonding, and their impact on shear bond strength and surface integrity at debonding.

**METHODS:**

Four experimental groups (n = 13) were set up according to the ceramic conditioning method: G1 = 37% phosphoric acid etching followed by silane application; G2 = 37% liquid phosphoric acid etching, no rinsing, followed by silane application; G3 = 10% hydrofluoric acid etching alone; and G4 = 10% hydrofluoric acid etching followed by silane application. After surface conditioning, metal brackets were bonded to porcelain by means of the Transbond XP system (3M Unitek). Samples were submitted to shear bond strength tests in a universal testing machine and the surfaces were later assessed with a microscope under 8 X magnification. ANOVA/Tukey tests were performed to establish the difference between groups (α= 5%).

**RESULTS:**

The highest shear bond strength values were found in groups G3 and G4 (22.01 ± 2.15 MPa and 22.83 ± 3.32 Mpa, respectively), followed by G1 (16.42 ± 3.61 MPa) and G2 (9.29 ± 1.95 MPa). As regards surface evaluation after bracket debonding, the use of liquid phosphoric acid followed by silane application (G2) produced the least damage to porcelain. When hydrofluoric acid and silane were applied, the risk of ceramic fracture increased.

**CONCLUSIONS:**

Acceptable levels of bond strength for clinical use were reached by all methods tested; however, liquid phosphoric acid etching followed by silane application (G2) resulted in the least damage to the ceramic surface.

## Abstract (pt)

**OBJETIVO:**

avaliar quatro métodos de condicionamento químico da superfície cerâmica, previamente à colagem de braquetes, e seu impacto sobre a resistência ao cisalhamento e a integridade da superfície ao descolamento.

**MÉTODOS:**

quatro grupos experimentais (n = 13) foram delineados de acordo com o método de condicionamento da superfície cerâmica empregado: G1 = ácido fosfórico a 37%, seguido da aplicação de silano; G2 = ácido fosfórico líquido a 37%, seguido da aplicação de silano sem lavagem prévia do ácido; G3 = ácido fluorídrico a 10%; e G4 = ácido fluorídrico a 10%, seguido da aplicação de silano. Após o condicionamento da superfície, braquetes metálicos foram colados à porcelana utilizando-se o sistema Transbond XP (3M Unitek). As amostras foram submetidas a ensaios de resistência ao cisalhamento, em máquina de ensaio universal, e as superfícies cerâmicas foram posteriormente avaliadas em microscópio, com magnitude de 8X. Testes ANOVA/Tukey foram realizados para verificar-se a diferença entre os grupos (α = 5%).

**RESULTADOS:**

os maiores valores de resistência ao cisalhamento foram encontrados nos grupos G_3_ e G_4_ (22,01 ± 2,15MPa e 22,83 ± 3,32Mpa, respectivamente), seguidos por G_1_ (16,42 ± 3,61MPa) e G_2_ (9,29 ± 1,95MPa). Quanto à avaliação da superfície após a descolagem do braquete, a utilização de ácido fosfórico líquido seguido da aplicação de silano, sem lavagem do ácido (G_2_), produziu menores danos à porcelana. Quando ácido fluorídrico e silano foram aplicados, o risco de fraturar a cerâmica aumentou.

**CONCLUSÕES:**

níveis aceitáveis de resistência de união para uso clínico foram alcançados por todos os métodos testados. No entanto, o condicionamento com ácido fosfórico líquido, seguido da aplicação de silano (G_2_), resultou em menor dano à superfície cerâmica.

## INTRODUCTION

The advent of adhesive systems has changed the technique of placing orthodontic appliances,^1^ thus enabling brackets to be bonded to anterior teeth and in the intermediate part of the arch, thereby replacing the system of bands previously used. This fact has overcome the main disadvantages of the multi-banded appliance, such as poor esthetics, clinical time spent on placement and need for individual tooth separation.

The quest for an esthetic smile and the extensive use of the bracket bonding technique aroused adult patients' interest in orthodontic treatment.^2^However, this context has posed a challenge to the bonding technique: the presence of artificial surfaces, since many adult patients have restorations that were performed with material such as composite resins, amalgams, gold, acrylic resin and/or porcelain.^2^^,^ 3 The demand for esthetics and technological advancements have caused the types of restorative material capable of accepting bracket bonding to increase, and a great variety of composites and ceramic systems are now available.^4^^,^ 5 Porcelain plays an important role in restorative systems, and it is used in veneers, inlays, onlays, full crowns and bridges. Porcelain good color stability provides an esthetic advantage over other restorative material; however, it is highly friable and its clinical repair does not yield satisfactory results.^2^^,^ 6

An adequate bonding technique implies that the bracket will support masticatory and orthodontic forces without being detached during treatment, thereby preserving the integrity of the tooth or restorative surface to be maintained after debonding.^3^ Surface conditioning is one of the most important factors in bracket bonding to the underlying artificial restorative surface. Ceramic surface conditioning can be performed by mechanical methods, such as increasing surface roughness by means of diamond burs, and air abrasion with aluminum oxide or silica; and chemical methods, such as acid etching, either with or without subsequent silane application.^7^^-^ 10

The choice between methods should take bracket bond strength and preservation of the ceramic surface after debonding into account. Adequate bond strength itself is not enough, if at the end of treatment the veneer or crown is damaged to the point where it needs to be replaced. However, preservation of the restorative work should not hamper adhesion or lead to successive rebonding. Therefore, appropriate bond strength should be allied to surface preservation.

It has been noted that chemical surface conditioning methods cause less damage to porcelain.^3^^,^ 10 ^,^ 11 ^,^ 12 Among the chemical methods assessed, the highest bond strength was observed when hydrofluoric acid was used, with or without subsequent silane application.^3^^,^ 4 ^,^ 6 ^,^ 10 ^,^ 13 ^-^ 17 However, the latter is capable of removing the glaze out of porcelain surface. Etching with 37% phosphoric acid followed by silane application was suggested as an alternative to hydrofluoric acid, but the acid should not be rinsed off between steps.^18^ This acid has the advantage of being routinely used in-office, in addition to being less aggressive to oral tissues and not removing the porcelain glaze.^18^

Therefore, the aim of this study was to assess four different chemical conditioning methods for porcelain surface before bracket bonding, as well as assess their impact over bond strength and surface integrity after debonding.

## MATERIAL AND METHODS

**Figure 1.**
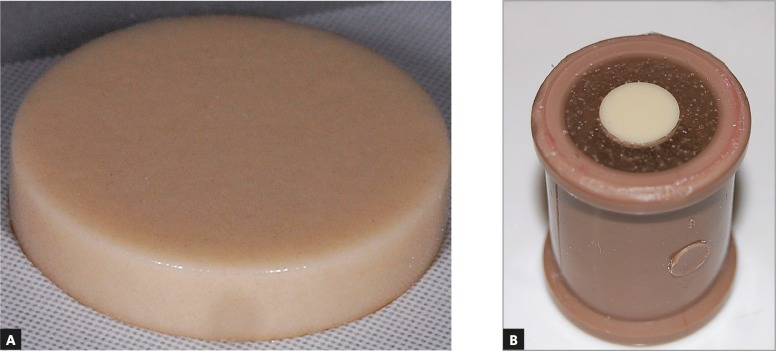
A) Feldspathic ceramic cylinder; B) Sample ready for the experiment.

This research had an experimental *in vitro* study design. Fifty-two feldspathic porcelain cylinders of the VITA VM13 system, (Wilcos do Brasil, Petrópolis/RJ, Brazil), 2.2 mm high and 10.4 mm wide, with glazed surfaces, were used in this study (Fig 1A).

The ceramic cylinders were inserted into PVC tubes filled with self-curing acrylic resin. During embedment, the ceramic samples were pressed against a wax sheet so that they remained stationary. Subsequently, the PVC tubes were placed near the ceramic disks, taking care to ensure that the samples remained centered. Finally, self-curing acrylic resin was poured into the tubes. Samples were ready for the experiment immediately after the acrylic resin was polymerized (Fig 1B).

All samples were polished with a rubber cup and fluoride-free pumice for 10 seconds, sprayed with water, and then dried with compressed oil-free air stream. They were randomly divided into four groups (n = 13), each one containing the number of samples obtained by sample size calculation for mean differences (α = 5%, study power = 80%), using data provided by Wang et al.^12^ Each experimental group underwent a different surface conditioning process (Table 1).

**Table 1.** Experimental group distribution according to each surface conditioning method.

Group	Surface conditioning method
G1	37% gel phosphoric acid etching for one minute (FGM, SC, Brazil), followed by water rinsing for another minute and air drying procedure. Silane application for one minute (Dentsply, Petrópolis, RJ, Brazil).
G2	37% liquid phosphoric acid etching for one minute (Reliance, Itasca, IL, USA), removal of excess acid with gentle air drying, followed by silane application for another minute (Dentsply, Petrópolis, RJ, Brazil). Surface was thoroughly washed and dried (Swartz,^18^ 2003).
G3	10% hydrofluoric acid etching for one minute (FGM, Joinville, SC, Brazil), followed by thorough washing and drying of surface.
G4	10% hydrofluoric acid etching for one minute (FGM, Joinville, SC, Brazil), followed by washing and drying of surface and application of silane for one minute (Dentsply, Petrópolis, RJ, Brazil).

The adhesive system used in this study was Transbond XT Light Cure Orthodontic Adhesive (3M Unitek, Monrovia, California, USA). After concluding the process of surface conditioning for each sample, adhesive was applied to the entire orthodontic bracket base. Standard Edgewise metal brackets for maxillary central incisors, with 80-micron mesh base (Morelli, Sorocaba/SP, Brazil), were chosen because their flat base allowed stable positioning on the samples. Each bracket was bonded to the center of the sample by means of applying a pressure of 260 gf. After bracket bonding, the adhesive was light-cured for 40 seconds (10 seconds on each bracket edge) using a calibrated LED Orthodontic Light activating device (Foshan Yunsheng Medical Instrument, Guangdong, China).

Each sample was submitted to shear bond strength test performed by a universal testing machine (EMIC DL2000, São José dos Pinhais, Brazil) at a speed of 0.5 mm/sec. The force per unit of area required to debond brackets was converted into megapascal (MPa) and named "shear bond strength." After debonding, the adhesive remnant index (ARI) was assessed under 10 x magnification (binocular optical microscope Nikon Eclipse E600, Nikon Corporation, Tokyo, Japan). Scores ranged from 0 to 3: 0 = absence of adhesive remnants; 1 = less than half of adhesive remnants; 2 = more than half of adhesive remnants; 3 = all adhesive remnants attached to the sample.^19^

A similar method was used to assess the damage caused to the ceramic surface. To this end, ceramic surface damage index (CSDI) was formulated, in which: 0 = no damage to the surface; 1 = absence of glaze on ceramic surface; 2 = presence of glaze and crack on ceramic surface; 3 = absence of glaze and presence of crack on ceramic surface; 4 = fractured ceramic surface. 

Statistical analyses were performed by means of SPSS version 16 (SPSS Inc., Chicago, Illinois, USA). Data were displayed in tables and submitted to descriptive analysis. Variables were checked for normality and homogeneity by means of Shapiro-Wilk and Levene tests, respectively; with significance level set at 0.05. Once normal and homogenous distribution of variables was established, analysis of variance (ANOVA) was performed to establish the difference between groups, followed by Tukey's multiple comparisons test.

## RESULTS

Table 2 displays descriptive analysis and ANOVA/Tukey tests results for the differences found in mean bond strength values. The ARI values are displayed in Table 3, whereas CSDI results are shown in Table 4.

**Table 2.** Shear bond strength values.

Groups	Mean bond strength (MPa)	Standard deviation	Maximum value	Minimum value	Statistical difference*
G1	16.42	3.61	22.49	9.43	a
G2	9.29	1.95	13.99	7.21	b
G3	22.01	2.15	26.62	17.98	c
G4	22.83	3.32	27.66	16.51	c

* Different letters suggest statistically significant differences as regards ANOVA/Tukey tests with significance level set at 0.05.

**Table 3.** ARI and fracture distribution per group.

	ARI				
	0	0	1	2	3
	With ceramic fracture	Without ceramic fracture			
G1 - n (%)	5 (38.46%)	5 (38.46%)	3 (23.07%)	0 (0%)	0 (0%)
G2 - n (%)	0 (0%)	9 (81.81%)	2 (18.18%)	0 (0%)	0 (0%)
G3 - n (%)	2 (16.66%)	6 (50%)	2 (16.66%)	0 (0%)	2 (16.66%)
G4 - n (%)	5 (38.46%)	2 (15.38%)	1 (7.,69%)	0 (0%)	5 (38.46%)

**Table 4.** Ceramic surface damage index (CSDI).

	Ceramic surface damage index				
	0 = none	1 = absence of glaze	2 = presence of glaze and crack	3 = absence of glaze and presence of crack	4 = fracture
G1 - n (%)	3 (23.07%)	1 (7,69%)	4 (30.76%)	0 (0%)	5 (38.46%)
G2 - n (%)	6 (54.54%)	0 (0%)	5 (45.45%)	0 (0%)	0 (0%)
G3 - n (%)	1 (8.33%)	0 (0%)	0 (0%)	9 (75%)	2 (16.66%)
G4 - n (%)	5 (38.46%)	0 (0%)	0 (0%)	3 (23.07)	5 (38.46%)

Groups G3 and G4, etched with hydrofluoric acid, yielded the highest shear bond strength values. The lowest values were found in G2 in which etching was performed with liquid phosphoric acid and subsequent silane application without previously rinsing the acid. 

The group in which the porcelain surface was found to be best preserved was G2 which presented no fractures.

## DISCUSSION

When orthodontic brackets are bonded to the enamel surface, bonding relies on adhesive penetration into the previously etched tooth surface and on formation of resin tags. In material with artificially glazed surfaces, such as porcelain, there is no such tag formation;^20^ for this reason, it demands different types of surface conditioning.

The shear bond strength results obtained in this study were 22.83 MPa, 22.02 MPa, 16.42 MPa and 9.29 MPa, pertaining to surfaces etched with 10% hydrofluoric acid and subsequent silane application (G4), 10% hydrofluoric acid alone (G3), 37% gel phosphoric acid and subsequent silane application (G1), and 37% liquid phosphoric acid and subsequent silane application without previously rinsing the acid (G2), respectively. As regards hydrofluoric acid etching, silane application was found to be unnecessary for direct bonding, which corroborates previous findings,^6^^,^ 21 since similar shear bond strength results were found.

When hydrofluoric acid was compared with phosphoric acid, higher shear bond strength values were found for the former, as previously observed.^11^^,^ 12 ^,^ 16 In spite of lower shear bond strength results being shown for phosphoric acid in comparison to hydrofluoric acid, the results proved to be clinically acceptable, and within the range of 6 to 8 Mpa established by Reynolds.^22^ It has been previously reported that phosphoric acid etching with subsequent silane application yields satisfactory clinical results in ceramic surface conditioning.^11^^,^ 12 ^,^ 18 ^,^ 23 ^,^ 24 ^,^ 25 According to Swartz,^18^ the use of liquid phosphoric acid and subsequent silane application without previously rinsing the acid causes acid and silane to interact on the ceramic surface. Thus, liquid acid must be used because if a gel etchant is applied, silane will be unable to reach the ceramic surface and react with it.

Phosphoric acid may be an alternative to hydrofluoric acid, since the latter is highly toxic and corrosive.^11^ Etching with 37% phosphoric acid for one minute will not scratch the porcelain, it will only clean the surface; and when used in association with silane, it reaches acceptable bond strength levels.^8^^,^ 11 ^,^ 12 ^,^ 16 ^,^ 23 ^-^ 26 Considering that phosphoric acid is routinely used in dental practice and knowing that its use in association with silane yields acceptable bond strength results for bracket bonding, this acid could be the first choice of material to be used for this procedure.^26^

ARI evaluation showed evidence of a marked concentration of samples that scored 0, with or without associated ceramic fracture; that is, a trend towards complete removal of adhesive together with the bracket under shear stress. Score 3 is considered the safest, in which adhesive remains completely attached to the sample after bracket debonding. Rotating burs are required to remove adhesive present after debonding, which should be done carefully in order to prevent removal of the glaze layer responsible for maintaining porcelain integrity and isolating cracks and porosities. It is imperative to maintain the material surface integrity; therefore, roughness should be avoided.^24^

CSDI showed that the least damage was observed in G2, as suggested by Swartz.^18^ Conversely, G4 was ranked highest in terms of percentage of fractured samples, supporting previous findings that claim more ceramic damage when hydrofluoric acid and silane are used in association.^11^^,^ 13 ^,^ 27 ^,^ 28 ^,^ 29 In spite of that, other authors have stated that surface etching with 10% hydrofluoric acid for 60 seconds and subsequent silane application represents no risk to the ceramic structure.^12^

When shear bond strength, ARI and CSDI values were analytically compared, the following was noted: G1 showed intermediate bond strength values meeting clinical requirements, absence of adhesive remnants on most of the sample surface, and moderate (presence of glaze and cracks on the surface) to slight (absence of glaze) damage to the ceramic surface; G2 showed the lowest shear bond strength values, although it also achieved minimal clinical requirements, the highest rate of surface preservation with 54.54% of samples left intact and 45.45% with only slight damage (presence of glaze and crack), and none of the samples fractured despite the high concentration of samples with score 0 in ARI; G3 showed good shear bond strength values, but with a high rate of surface damage, with 16.66% of samples presenting fractures, and 75% of them presenting cracks and lack of glaze; G4 also showed high bond strength values, but similarly to G3, there were high levels of surface fracture (38.46%) and lack of glaze and cracking in 27.07% of cases.

Whenever preparing porcelain surfaces for bracket bonding, one should take all aforementioned characteristics into account. Thus, liquid phosphoric acid with subsequent silane application without previously rinsing the acid (G2), as described by Swartz,^18^ seems to have yielded the best combination of results in terms of ARI, CSDI and shear bond strength values. This method was initially described over 10 years ago, but has not been widely used in *in vitro* studies or in the orthodontic practice.

One of the limitations of the present study is related to sample storage. The high shear bond strength results and surface damage may be related to lack of thermal cycling which, according to the literature, can increase shear bond strength and ceramic damage.^14^^,^ 30 *Future research should perform thermal cycling of samples before shear bond strength testing.*

The results of this research should be considered for clinical application with caution, as it is a laboratory study. Clinical tests are also rendered necessary.

## CONCLUSIONS

The highest shear bond strength values were obtained when hydrofluoric acid was used, with or without subsequent silane application (G3 and G4); whereas the lowest values were found when surfaces were etched with liquid phosphoric acid with subsequent drying and silane application (G2). However, all surface conditioning methods seem to result in acceptable bond strength levels.

Liquid phosphoric acid and subsequent silane application (G2) caused the least damage to the ceramic surface. As regards hydrofluoric acid etching, subsequent silane application seemed to have increased the risk of ceramic surface fracture.

## References

[1] Fox NA, McCabe JF, Buckley JG (1994). A critique of bond strength testing in orthodontics. Br J Orthod.

[2] Winchester L (1991). Direct orthodontic bonding to porcelain: an in vitro
study. Br J Orthod.

[3] Akova T, Yoldas O, Toroglu MS, Uysal H (2005). Porcelain surface treatment by laser for bracket-porcelain
bonding. Am J Orthod Dentofacial Orthop.

[4] Türk T, Saraç D, Saraç YS, Elekdag-Türk S (2006). Effects of surface conditioning on bond strength of metal brackets to
all-ceramic surfaces. Eur J Orthod.

[5] Leinfelder KF (2000). Porcelain esthetics for the 21st century. J Am Dent Assoc.

[6] Schmage P, Nergiz I, Herrmann W, Ozcan M (2003). Influence of various surface-conditioning methods on the bond strength
of metal brackets to ceramic surfaces. Am J Orthod Dentofacial Orthop.

[7] Matinlinna JP, Lassila LV, Ozcan M, Yli-Urpo A, Vallittu PK (2004). An introduction to silanes and their clinical applications in
dentistry. Int J Prosthodont.

[8] Ajlouni R, Bishara SE, Oonsombat C, Soliman M, Laffoon J (2005). The effect of porcelain surface conditioning on bonding orthodontic
brackets. Angle Orthod.

[9] Goyal S (2006). Silanes: Chemistry and applications. J Indian Prosthodont Soc.

[10] Gillis I, Redlich M (1998). The effect of different porcelain conditioning techniques on shear
bond strength of stainless steel brackets. Am J Orthod Dentofacial Orthop.

[11] Bourke BM, Rock WP (1999). Factors affecting the shear bond strength of orthodontic brackets to
porcelain. Br J Orthod.

[12] Wang H, Xiong F, Zhenhua L (2011). Influence of varied surface texture of dentin porcelain on optical
properties of porcelain specimens. J Prosthet Dent.

[13] Chung CH, Brendlinger EJ, Brendlinger DL, Bernal V, Mante FK (1999). Shear bond strengths of two resin-modified glass ionomer cements to
porcelain. Am J Orthod Dentofacial Orthop.

[14] Huang TH, Kao CT (2001). The shear bond strength of composite brackets on porcelain
teeth. Eur J Orthod.

[15] Harari D, Shapira-Davis S, Gillis I, Roman I, Redlich M (2003). Tensile bond strength of ceramic brackets bonded to porcelain
facets. Am J Orthod Dentofacial Orthop.

[16] Larmour CJ, Bateman G, Stirrups DR (2006). An investigation into the bonding of orthodontic attachments to
porcelain. Eur J Orthod.

[17] Imakami MB, Valle-Corotti KM, Carvalho PEG, Scocate ACRN (2011). Evaluation of shear strength of lingual brackets bonded to ceramic
surfaces. Dental Press J Orthod.

[18] Swartz ML (2003). Reliable porcelain bonding. Clin Impress.

[19] Artun J, Bergland S (1984). Clinical trials with crystal growth conditioning as an alternative to
acid-etch enamel pretreatment. Am J Orthod.

[20] Smith GA, McInnes-Ledoux P, Ledoux WR, Weinberg R (1988). Orthodontic bonding to porcelain: bond strength and
refinishing. Am J Orthod Dentofacial Orthop.

[21] Zachrisson BU (2000). Orthodontic bonding to artificial tooth surfaces: clinical versus
laboratory findings. Am J Orthod Dentofacial Orthop.

[22] Reynolds IR (1975). A review of direct orthodontic bonding. Br J Orthod.

[23] Whitlock 3rd BO, Eick JD, Ackerman RJ, Glaros AG, Chappell RP (1994). Shear strength of ceramic brackets bonded to porcelain. Am J Orthod Dentofacial Orthop.

[24] Nebbe B, Stein E (1996). Orthodontic brackets bonded to glazed and deglazed porcelain
surfaces. Am J Orthod Dentofacial Orthop.

[25] Pannes DD, Bailey DK, Thompson JY, Pietz DM (2003). Orthodontic bonding to porcelain: a comparison of bonding
systems. J Prosthet Dent.

[26] Aboush YE (1998). Removing saliva contamination from porcelain veneers before
bonding. J Prosthet Dent.

[27] Cochran D, O'Keefe KL, Turner DT, Powers JM (1997). Bond strength of orthodontic composite cement to treated
porcelain. Am J Orthod Dentofacial Orthop.

[28] Falkensammer F, Freudenthaler J, Pseiner B, Bantleon HP (2012). Influence of surface conditioning on ceramic microstructure and
bracket adhesion. Eur J Orthod.

[29] Ramos TF, Lenza MA, Reges RR, Freitas G (2012). Influence of ceramic surface treatment on shear bond strength of
ceramic brackets. Indian J Dent Res.

[30] Zachrisson YO, Zachrisson BU, Büyükyilmaz T (1996). Surface preparation for orthodontic bonding to
porcelain. Am J Orthod Dentofacial Orthop.

